# Double knockdown of α1,6-fucosyltransferase (*FUT8*) and GDP-mannose 4,6-dehydratase (*GMD*) in antibody-producing cells: a new strategy for generating fully non-fucosylated therapeutic antibodies with enhanced ADCC

**DOI:** 10.1186/1472-6750-7-84

**Published:** 2007-11-30

**Authors:** Harue Imai-Nishiya, Katsuhiro Mori, Miho Inoue, Masako Wakitani, Shigeru Iida, Kenya Shitara, Mitsuo Satoh

**Affiliations:** 1 Tokyo Research Laboratories, Kyowa Hakko Kogyo Co., Ltd., 3-6-6 Asahi-machi, Machida-shi, Tokyo 194-8533, Japan

## Abstract

**Background:**

Antibody-dependent cellular cytotoxicity (ADCC) is greatly enhanced by the absence of the core fucose of oligosaccharides attached to the Fc, and is closely related to the clinical efficacy of anticancer activity in humans *in vivo*. Unfortunately, all licensed therapeutic antibodies and almost all currently-developed therapeutic antibodies are heavily fucosylated and fail to optimize ADCC, which leads to a large dose requirement at a very high cost for the administration of antibody therapy to cancer patients. In this study, we explored the possibility of converting already-established antibody-producing cells to cells that produce antibodies fully lacking core fucosylation in order to facilitate the rapid development of next-generation therapeutic antibodies.

**Results:**

Firstly, loss-of-function analyses using small interfering RNAs (siRNAs) against the three key genes involved in oligosaccharide fucose modification, i.e. α1,6-fucosyltransferase (*FUT8*), GDP-mannose 4,6-dehydratase (*GMD*), and GDP-fucose transporter (*GFT*), revealed that single-gene knockdown of each target was insufficient to completely defucosylate the products in antibody-producing cells, even though the most effective siRNA (>90% depression of the target mRNA) was employed. Interestingly, beyond our expectations, synergistic effects of *FUT8 *and *GMD *siRNAs on the reduction in fucosylation were observed, but not when these were used in combination with *GFT *siRNA. Secondly, we successfully developed an effective short hairpin siRNA tandem expression vector that facilitated the double knockdown of *FUT8 *and *GMD*, and we converted antibody-producing Chinese hamster ovary (CHO) cells to fully non-fucosylated antibody producers within two months, and with high converting frequency. Finally, the stable manufacture of fully non-fucosylated antibodies with enhanced ADCC was confirmed using the converted cells in serum-free fed-batch culture.

**Conclusion:**

Our results suggest that FUT8 and GMD collaborate synergistically in the process of intracellular oligosaccharide fucosylation. We also demonstrated that double knockdown of *FUT8 *and *GMD *in antibody-producing cells could serve as a new strategy for producing next-generation therapeutic antibodies fully lacking core fucosylation and with enhanced ADCC. This approach offers tremendous cost- and time-sparing advantages for the development of next-generation therapeutic antibodies.

## Background

Antibodies of the human IgG1 isotype containing two biantennary complex-type *N*-linked oligosaccharides in the constant region (Fc) [1] are commonly used therapeutically. As regards cancer treatment in particular, the antibody effector function of antibody-dependent cellular cytotoxicity (ADCC) is known to be important and is closely related to clinical efficacy in humans *in vivo *[2-4]. Through the Fc, therapeutic antibodies can mediate effector functions, and ADCC is greatly influenced by Fc oligosaccharide structure [5,6]. Removal of the core fucose from Fc oligosaccharides is widely recognized as being important for the effector function of ADCC [7,8]. Antibodies in which the Fc oligosaccharide structure lacks the core fucose exhibit more potent efficacy than do fucosylated antibodies, both *in vitro *and *in vivo *[9-13]. Therapeutic antibodies fully lacking core fucosylation are able to escape the inhibitory effects of both human serum IgG and other contaminating fucosylated antibody ingredients to achieve optimal ADCC [6,14-17]. Unfortunately, almost all licensed therapeutic antibodies developed to date are heavily fucosylated, i.e., the majority of antibody molecules possess Fc oligosaccharides with the core fucose [18,19], which results in a failure to optimize ADCC. The presence of this core fucose is largely due to the fact that the antibodies are produced by rodent mammalian cell lines with intrinsic fucosyltransferase activity (e.g., Chinese hamster ovary (CHO), mouse myeloma NS0 and SP2/0, and mouse hybridoma cell lines).

**Figure 1:**
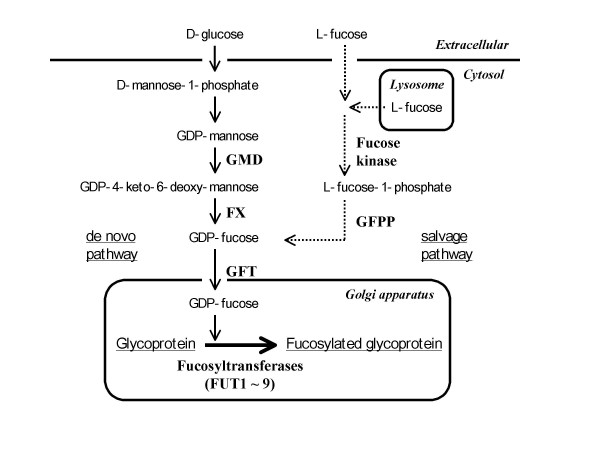
**Oligosaccharide fucosylation and GDP-fucose synthesis in mammalian cells**. In mammalian cells, GDP-fucose is synthesized via two distinct pathways, the *de novo *and salvage pathways. The transport of GDP-fucose into the Golgi apparatus, where the fucosyltransferases are located, is accomplished by a GDP-fucose transporter.

In mammalian cells, core fucosylation of the Fc oligosaccharides is mediated by the only gene, α1,6-fucosyltransferase (*FUT8*), that catalyzes the transfer of fucose from GDP-fucose to the innermost *N*-acetylglucosamine (GlcNAc) of Fc oligosaccharides via an α1,6-linkage [20]. The intracellular GDP-fucose, an essential substrate of oligosaccharide fucosylation, is synthesized in the cytoplasm via both a *de novo *pathway and the salvage pathway shown in Fig. 1. The *de novo *pathway transforms GDP-mannose, which originates from D-glucose taken into the cytoplasm from the extracellular environment, to GDP-fucose, via three enzymatic reactions carried out by two proteins: GDP-mannose 4,6-dehydratase (GMD) and GDP-keo-6-deoxymannose 3,5-epimerase, 4-reductase (FX) [21,22]. The salvage pathway synthesizes GDP-fucose from free L-fucose derived from extracellular or lysosomal sources. Most of the intracellular GDP-fucose is generated via the *de novo *pathway, and the metabolite-free L-fucose is also reutilized through the salvage pathway [22]. The GDP-fucose, which accumulates in the cytoplasm, is transported into the lumen of the Golgi apparatus by a GDP-fucose transporter (GFT) anchored at the Golgi membrane [23], and then serves as a substrate in the synthesis of fucosylated glycoconjugates by fucosyltransferases [22,24,25].

To date, only a few studies have addressed the regulation of Fc oligosaccharide fucosylation in mammalian cells using the following approaches: 1) the application of a mutant CHO cell line, Lec13, partially deficient in *GMD *[7] or that of a rat hybridoma cell line, YB2/0 [8], as host cells; 2) the introduction of a small interfering RNA (siRNA) against *FUT8 *[26]; and 3) the co-expression of β-1,4-*N*-acetylglucosaminyltransferase III (*GnT-III*) and Golgi α-mannosidase II (*ManII*) [27]. Of these, the gene knockout of *FUT8 *and *GMD *is the only strategy for manufacturing fully non-fucosylated recombinant therapeutics in mammalian cells [28-30]. However, gene targeting in mammalian somatic cells is difficult to achieve and is a laborious and time-consuming process, because in somatic cells, non-homologous recombination events occur several orders of magnitude more frequently than homologous recombination [28,31]. Thus, gene targeting in mammalian somatic cells remains very difficult to apply to each antibody-producing clone as a simple means of controlling fucosylation.

In this study, we explored the possibility of the high-frequency conversion of already-established antibody-producing cells (1^st^-generation) to cells that produce antibodies fully lacking core fucosylation (2^nd^-generation) within a few months. Moreover, it was considered industrially useful to generate 2^nd^-generation cells that would yield the equivalent antibody productivity of the original cells, without inducing any changes in cell character beyond the lack of fucosylation. To this end, we applied an RNA interference (RNAi) technique in combination with a cellular phenotypic selection strategy using *Lens culinaris *agglutinin (LCA) lectin, which recognizes the α1,6 fucosylated trimannose-core structure of *N*-linked oligosaccharides and commits cells expressing this structure to a cell-death pathway. In our previous study, single-gene knockdown of *FUT8 *resulted in a substantial, but not full, reduction of antibody fucosylation in the products [26]. Here, we identified synergistic effects of the double knockdown of *FUT8 *and *GMD *on oligosaccharide fucosylation in mammalian cells. This finding enabled us to design a new conversion strategy for the manufacture of next-generation therapeutic antibodies fully lacking core fucosylation and with enhanced ADCC.

## Results

### Single gene knockdown of *FUT8*, *GMD*, and *GFT *in antibody-producing cells

Three constitutive short hairpin siRNA expression vectors against Chinese hamster *FUT8*, *GMD*, and *GFT *were generated and introduced into an IgG1 antibody-producing CHO/DG44 clone, 32-05-12 [26], to evaluate the effects of target gene knockdown on the levels of fucosylation of the products. Puromycin-resistant clones transformed by the vectors appeared after transfection with a transformation efficiency of approximately 1,300 per 1.6 × 10^6 ^electroporated cells, irrespective of the vector differences. However, subsequent selection of LCA-resistant clones showed clear differences in the appearance of surviving colonies. The ratios of LCA-resistant clones to puromycin-resistant clones transformed by the three siRNA expression vectors against *FUT8*, *GMD*, and *GFT *were 11.5%, 14.8%, and 0.8%, respectively (Table 1). The resultant LCA-resistant clones were expanded in medium without LCA, and the mRNA expression of *FUT8*, *GMD*, and *GFT*, as well as the oligosaccharide structure of the antibodies produced in these clones, were quantitatively analyzed. The target gene mRNA was reduced to a level exceeding 90% in clones appearing in all three siRNA-introduced transformants; however, in none of the clones was Fc oligosaccharide fucosylation completely abolished. The most effective rates of reduction in fucosylation among the clones transformed by the *FUT8*, *GMD*, and *GFT *siRNA expression vectors were approximately 72%, 79%, and 42%, respectively.

**Table 1:** Ratio of LCA-resistant clones to drug-resistant clones transformed by the introduction of siRNA expression vectors

Vector	Target mRNA	Drug^r ^clones^a^	LCA^r ^clones^b^	% LCA^r^/Drug^r^
FUT8shRNA/lib3/pPUR	*FUT8*	1344	155	11.5
pPUR/GMDshB	*GMD*	1440	213	14.8
GFT_3G10/pPUR	*GFT*	1296	11	0.8
FT8lib3_GMDB/pAGE	*GMD·FUT8*	1500	730	48.7

^a^Drug^r^, puromycin resistance or hygromycin resistance.

^b^LCA^r^, LCA resistance

### Synergistic effects of double knockdown of *FUT8 *and *GMD *on fucosylation

To explore the effects of double knockdown of the target genes *FUT8 *and *GMD*, two *GMD *siRNA-introduced clones exhibiting a maximum reduction in product fucosylation, designated as GMDb2 and GMDb5, were selected and re-electroporated with the *FUT8 *siRNA expression vector. LCA-resistant clones were selected in the presence of 100 μM L-fucose after drug-resistant selection had been carried out, and two clones expressing highly non-fucosylated antibodies were selected independently from each *GMD *siRNA-introduced clone in the medium. In the four established clones, the *FUT8 *mRNA expression levels decreased to roughly one-tenth of the parental expression levels, while the original 90% decrease in *GMD *mRNA was retained. Monosaccharide composition analysis showed that the ratio of non-fucosylated oligosaccharides of the antibodies produced by the four clones had increased significantly in each case (i.e., up to 91%, 95%, 97%, and 98%), compared to those of parental *GMD *siRNA-introduced clones (Table 2). The increase in non-fucosylated product levels was cancelled when the clones were cultured in the presence of L-fucose; this cancellation was ascribed to the absence of a *GMD *knockdown effect due to activation of the salvage pathway of GDP-fucose synthesis. No significant effect on fucosylation levels in antibody-producing cells was observed with a combination of either *FUT8 *and *GFT *or *GMD *and *GFT *double knockdown.

**Table 2:** Monosaccharide composition of the *N*-linked oligosaccharide core structure of IgG1 from clones transformed with siRNA expression vectors

Clone Name	siRNA^a^	LF^b^	Relative composition of monosaccharide			Fucose(-)%^d^
				
			Fucose^c^	GlcNAc	Mannose	
32-05-12	-	-	0.97	4.00	2.67	3
GMDb2	GMD	-	0.22	4.00	2.52	78
GMDb5	GMD	-	0.21	4.00	2.53	79
GMDb2-F3	GMD·FUT8	-	0.02	4.00	2.70	98
GMDb2-F5	GMD·FUT8	-	0.03	4.00	2.71	97
GMDb5-F2	GMD·FUT8	-	0.05	4.00	2.77	95
GMDb5-F4	GMD·FUT8	-	0.09	4.00	2.70	91
32-05-12	-	+	0.94	4.00	2.56	6
GMDb2	GMD	+	0.94	4.00	2.56	6
GMDb5	GMD	+	0.92	4.00	2.60	8
GMDb2-F3	GMD·FUT8	+	0.19	4.00	2.52	81
GMDb2-F5	GMD·FUT8	+	0.21	4.00	2.54	79
GMDb5-F2	GMD·FUT8	+	0.19	4.00	2.55	81
GMDb5-F4	GMD·FUT8	+	0.33	4.00	2.68	67

^a^GMD, introduction of *GMD *siRNA expression vector; GMD·FUT8, introduction of *FUT8 *siRNA expression vector to *GMD *siRNA-introduced cells.

^b^LF, addition of 100 μM L-fucose into the culture medium.

^c^Molar ratios calculated vs. 4 GlcNAc.

^d^Total percentage of non-fucosylated Fc oligosaccharides calculated by the formula (1-c) × 100.

### Conversion of fucosylated antibody-producing cells to non-fucosylated antibody producers

**Figure 2:**
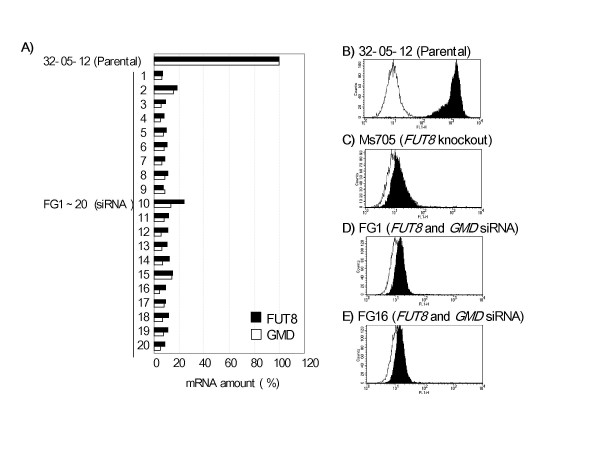
**Analyses of clones transformed by the *FUT8 *and *GMD *siRNA tandem expression vector**. The relative amounts of *FUT8 *(filled columns) and *GMD *(open columns) mRNA were quantified in the tandem siRNA expression vector-introduced LCA-resistant cells and parental cells (A). Each cell type was harvested after 3-day culture and the samples were analyzed by real-time PCR. Each mRNA amount was normalized to the amount of β-actin mRNA; the results are shown as the relative percent with respect to the parental cells (100%). The LCA reactivity of cells (32-05-12 (B), Ms705 (C), FG1 (D), and FG16 (E)) was analyzed after adaptation of the cells to serum-free medium. Each cell type was harvested after 6-day culture and stained with FITC-labeled LCA (filled peak) or FITC-labeled streptavidin (open peak) as a negative control, and the results were analyzed by FACS.

To facilitate the double knockdown of *FUT8 *and *GMD*, the siRNA tandem expression vector for targeting these genes was generated and introduced into IgG1 antibody-producing CHO/DG44 32-05-12 cells. Hygromycin-resistant clones transformed by the vectors appeared with a common transformation efficiency of approximately 1,500 per 1.6 × 10^6 ^electroporated cells. However, subsequent selection of LCA-resistant clones revealed clear differences in the appearance of surviving colonies, as compared to that of single siRNA-introduced clones (Table 1). The ratio of LCA-resistant clones to hygromycin-resistant clones transformed by the siRNA tandem expression vector was much higher (more than triple) than that of clones transformed by the vector bearing each single siRNA expression cassette; approximately half of the hygromycin-resistant clones showed resistance to LCA, even in the culture condition containing L-fucose. Among the resultant LCA-resistant clones, twenty were randomly selected and expanded in medium lacking both LCA and L-fucose. The mRNA expression of both target genes, *FUT8 *and *GMD*, in each clone decreased to approximately less than 20% of that of the parental clone (Fig. 2A). Out of twenty clones, eight exhibited relatively low levels of both target genes, and these were selected for further analysis of the oligosaccharide structure of the antibody products. The Fc oligosaccharide structures of the antibodies produced by these eight clones showed almost complete non-fucosylation; there were no detectable L-fucose residues among the products from six clones (Table 3). These six clones exhibited very low reactivity to LCA, even after adaptation to serum-free medium, as was also observed in the case of the *FUT8*-knockout CHO cell line Ms705. The results obtained from two representative clones, designated as FG1 and FG16, are shown in Fig. 2B–2E.

**Table 3:** Monosaccharide composition of the *N*-linked oligosaccharide core structure of IgG1 from clones transformed with the *FUT8 *and *GMD *siRNA tandem expression vector

Clone Name	siRNA^a^	Relative composition of monosaccharide			Fucose(-)%^c^
			
		Fucose^b^	GlcNAc	Mannose	
32-05-12	-	0.96	4.00	2.60	4
FG1	+	n.d.	4.00	2.58	100
FG3	+	n.d.	4.00	2.65	100
FG4	+	n.d.	4.00	2.63	100
FG7	+	0.02	4.00	2.57	98
FG9	+	n.d.	4.00	2.61	100
FG13	+	0.02	4.00	2.55	98
FG16	+	n.d.	4.00	2.57	100
FG20	+	n.d.	4.00	2.62	100

^a^Introduction of *FUT8 *and *GMD *siRNA tandem expression vector.

^b^Molar ratios calculated vs. 4 GlcNAc; n.d., not detectable.

^c^Total percentage of non-fucosylated Fc oligosaccharides calculated by the formula (1-b) × 100.

### Serum-free fed-batch culture of siRNA-introduced cells producing non-fucosylated antibodies

**Figure 3:**
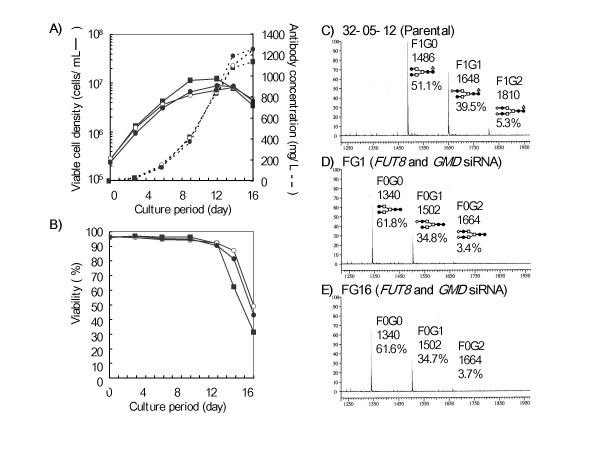
**Serum-free fed-batch culture of clones transformed by the *FUT8 *and *GMD *tandem siRNA expression vector**. Serum-free fed-batch culture using a 1L spinner bioreactor was carried out using cells transformed by the *FUT8 *and *GMD *tandem siRNA expression vector (FG1 (open circles) and FG16 (filled circles)). The parental cell line 32-05-12 (filled squares) was cultured as a control. Viable cell density (A, solid lines), antibody concentration in the culture supernatant (A, dotted lines), and cell viability (B) were analyzed in the fed-batch culture. The oligosaccharide structures of the final products from 32-05-12 (C), FG1 (D), and FG16 (E) were analyzed using MALDI-TOF MS. The relative composition of each peak is shown as the relative amount to the total amount of oligosaccharide detected.

**Figure 4:**
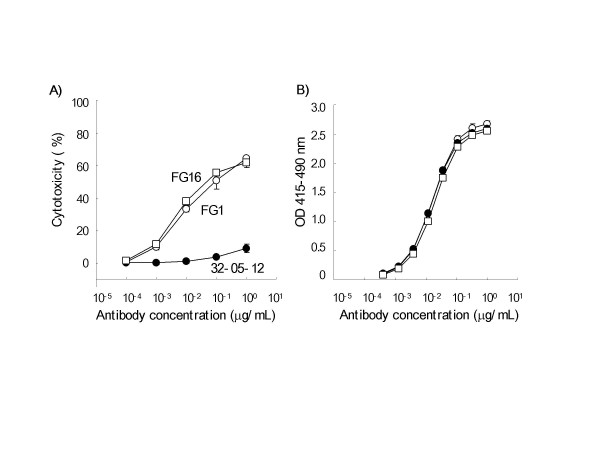
**Biological activity of antibodies from clones transformed with the *FUT8 *and *GMD *siRNA tandem expression vector**. Lysis of antigen-expressing cells targeted by human PBMCs at a target:effector ratio of 1:20 in the presence of different antibody concentrations was quantified by detecting lactate dehydrogenase activity (A). The antigen-binding activity of the antibody was measured by ELISA (B). Antibody purified from the serum-free fed-batch cultures of cells transformed by the *FUT8 *and *GMD *tandem siRNA expression vector, FG1 (open circles), FG16 (open squares), and the parental cell line 32-05-12 (filled circles) are shown. Cytotoxicity (%) and absorbance are indicated as the mean values ± SD of triplicates.

Serum-free fed-batch culture of the clones transformed by the *FUT8 *and *GMD *siRNA tandem expression vector was carried out using 1L-scale spinner bioreactors with pH and DO controls. Two *FUT8 *and *GMD *siRNA-introduced clones, FG1 and FG16, and their parental IgG1-producing clone, CHO/DG44 32-05-12, were adapted to serum-free medium, and the performance of the fed-batch cultures was compared in a head-to-head analysis (Fig. 3A, 3B). The fed-batch cultures were maintained until the cell viability decreased to less than 50%, which occurred at day 16 post-inoculation. Culture aliquots were taken at days 3, 6, 9, 12, 14, and 16 to analyze the cells and antibody products. Two siRNA-introduced cell lines grew logarithmically, with a slight difference in the specific production rate, maximum viable cell density, and the day upon which the viable cell density reached the maximum level; two siRNA-introduced hygromycin-resistant descendants showed slightly slower growth and death rates than those of the parent cells. However, the productivity of two clones transformed by *FUT8 *and *GMD *siRNAs reached a production level of approximately 1200 mg/L, which was basically equivalent to that of the parental cells at the end of the fed-batch culture period. During the culture period, the levels of cellular *FUT8 *and *GMD *mRNAs in the two siRNA-introduced clones remained at the original low level, i.e., less than 20% of the parental level. Monosaccharide composition analysis revealed that the two siRNA-introduced clones stably produced non-fucosylated antibodies throughout the culture period (Table 4). The oligosaccharide profile analysis of products purified from the final culture medium confirmed that the *N*-linked Fc oligosaccharides of the products were of the biantennary complex type, and the products from the two siRNA-introduced clones fully lacked core fucosylation (Fig. 3D, 3E). It was also confirmed that there was no significant change in the oligosaccharide profile during the culture period, and the non-fucosylated products showed two orders of magnitude higher ADCC than the antibodies from the parental CHO/DG44 cells, without any changes in antigen binding (Fig. 4).

**Table 4:** Monosaccharide composition of the *N*-linked oligosaccharide core structure of IgG1 from clones transformed with the *FUT8 *and *GMD *siRNA tandem expression vector during fed-batch culture

Clone Name	Culture period (day)	Relative composition of monosaccharide			Fucose(-)%^b^
			
		Fucose^a^	GlcNAc	Mannose	
32-05-12	6	0.92	4.00	2.86	8
	12	0.94	4.00	2.99	6
	16	0.97	4.00	3.01	3
FG1	6	n.d.	4.00	2.85	100
	12	n.d.	4.00	2.97	100
	16	n.d.	4.00	2.92	100
FG16	6	n.d.	4.00	2.82	100
	12	n.d.	4.00	2.96	100
	16	n.d.	4.00	2.96	100

^a^Molar ratios calculated vs. 4 GlcNAc; n.d., not detectable.

^b^Total percentage of non-fucosylated Fc oligosaccharides calculated by the formula (1-a) × 100.

## Discussion

The conversion of fucosylated therapeutic antibodies to antibodies lacking the core fucose in the Fc is recognized as an attractive approach for generating next-generation therapeutics. In this study, we explored the possibility of the high-frequency conversion of already-established antibody-producing cells (1^st^-generation) to cells that produce antibodies fully lacking core fucosylation (2^nd^-generation) within a few months, while almost all original features of the 1^st^-generation antibody-producing cells, including cell growth and recombinant protein productivity, were retained. The present approach is expected to be industrially useful for the establishment of a master cell bank of antibody-producing cells for the rapid development of 2^nd^-generation therapeutic antibodies.

Here, we focused on three key genes involved in the oligosaccharide fucosylation pathway in mammalian cells (*FUT8*, *GMD*, and *GFT*), and we identified an effective siRNA of each gene among more than ten siRNA candidates using a transient expression system with a target gene-GFP fusion reporter construct, as previously reported (data not shown) [32]. Every identified siRNA showed high efficacy in terms of depressing the target mRNA to levels of over 90%, when it was expressed in the form of short hairpin siRNA under the control of U6 or the tRNA^val ^promoter in antibody-producing cells in which the siRNA expression unit was integrated in the genome. The present results thus demonstrated that our established siRNA expression system was able to sufficiently decrease level of target mRNA to the current maximum level achievable with RNAi technology. The levels of reduction in Fc fucosylation among the products were found to vary, depending on both target gene and clone, even when specific target genes were reduced to comparable levels in the siRNA-introduced cells. Cellular phenotypic selection of resistance to LCA was conducted to enrich transformants with cellular features equivalent to those of *FUT8*-knockout cells. However, among clones transformed by either siRNA alone, none produced fully lacking the core fucose of the Fc. These results suggest that there is a fucosylation-reducing limit associated with single-gene knockdown of *FUT8*, *GMD*, and *GFT *using RNAi technology.

On the other hand, we clearly observed a noteworthy synergistic effect of double knockdown of *FUT8 *and *GMD *on the fucosylation of products in antibody-producing cells (Table 2), although neither the combination of *FUT8 *and *GFT*, nor *GMD *and *GFT *double knockdown caused a significant change. This synergistic effect was not accounted for by the levels of mRNA regulation of target genes, because no additive effect on the level of depression of each target mRNA was observed with the *FUT8 *and *GMD *double knockdown. Some previous studies have shown that there is no functional redundancy in FUT8 and GMD, although there is a fuctional redundancy in GFT [28,30,33,34]. The loss of *GFT *seemed to be compensated for by other genes possessing transporter activity. In ordinary cell cultures, *FUT8 *is considered to be the only rate-limiting step enzyme of fucosylation, since mammalian cells retain excess endogenous intracellular GDP-fucose [30,35]. In theory, the level of product fucosylation should be controlled, either in a manner dependent on endogenous FUT8 activity under cellular conditions of abundant intracellular GDP-fucose, or in a manner dependent on endogenous GMD activity under condition of intracellular GDP-fucose starvation; these genes are not currently believed to act in an interdependent manner. However, the present results clearly demonstrated that residual FUT8 activity in single *FUT8*-gene knockdown in mammalian CHO cells with a reduced substrate supply due to a second *GMD*-gene knockdown yields an even lower rate of fucose transfer to the oligosaccharides of the products than that achieved with single *FUT8*-gene knockdown cells. The intracellular GDP-fucose concentration in the Golgi apparatus might have an effect on the function of FUT8, although the precise reasons for the observed synergy of *FUT8 *and *GMD *double knockdown in terms of reducing intracellular oligosaccharide fucosylation remain unclear.

Single-step conversion of already-established antibody-producing cells to non-fucosylated antibody producers by double knockdown of *FUT8 *and *GMD *was attempted in order to verify the industrial applicability of such a system. Our transformation strategy was found to function quite well; in brief, we introduced an siRNA tandem expression vector for introducing the double knockdown of *FUT8 *and *GMD *into antibody-producing cells, and we carried out the cellular phenotypic selection of resistance to LCA in the presence of L-fucose. We successfully established antibody-producing clones in which no fucosylated oligosaccharides were detected by either monosaccharide composition or MALDI-TOF MS analyses (Table 3, 4, Fig. 3D, 3E). Due to the very high conversion frequency achieved with this approach, only two months were required to complete all steps of the conversion; the ratio of LCA-resistant clones to drug-resistant clones transformed by the siRNA tandem expression vector was higher than that of clones transformed by each single siRNA expression unit (Table 1), and six clones out of the twenty LCA-resistant transformants randomly selected were found to be converted precisely to the desired clones (Table 3). The established converted clones proved capable of consistently producing fully non-fucosylated antibodies throughout the duration of a serum-free fed-batch culture period; furthermore, a constant ratio of F0G0, F0G1, and F0G2 oligosaccharides was maintained until the end of the culture period (Fig. 3, Table 4). The antibody productivity of the clones was also found to be equivalent to that of the parental cells at the end of the culture period. The purified antibodies obtained from the cultures exhibited approximately 100-fold higher ADCC compared to that of the original antibodies obtained from the parental cells (Fig. 4). The stability of the converted clones was confirmed for a long culture period of over 30 passages with stable integration of the siRNA expression unit into the genome (data not shown). The production and purification processes established for the 1^st^-generation therapeutic antibodies could be applied, with only marginal modifications, to establish the 2^nd^-generation processes. Thus, the present approach could provide substantial time and cost benefits for the development of next-generation therapeutic antibodies. Our preliminary experiments have also indicated that the tandem siRNA expression vector designed for the double knockdown of mouse *FUT*8 and *GMD *also worked in mouse myeloma cell lines NS0 and SP2/0.

## Conclusion

The double knockdown of *FUT8 *and *GMD *in antibody-producing cells is worth considering as a novel strategy for generating therapeutic antibodies fully lacking core fucosylation with enhanced ADCC. The tremendous cost- and time-sparing advantages of this approach are expected to facilitate the development of next-generation therapeutic antibodies. This approach is based on our observation of the synergistic effects of double knockdown of *FUT8 *and *GMD *on intracellular oligosaccharide fucose modification in mammalian cells.

## Methods

### Cell lines

The dihydrofolate reductase-deficient CHO cell line, CHO/DG44 [36], was obtained from Dr. Lawrence Chasin of Columbia University (New York). A recombinant mouse/human chimeric IgG1-producing CHO/DG44 cell line, 32-05-12, generated as described previously [26], was cultured in IMDM medium (Invitrogen, Carlsbad, CA) containing 10% (v/v) dialyzed fetal bovine serum (dFBS; Invitrogen) and 500 nM methotrexate (MTX; Sigma-Aldrich, St. Louis, MO).

### Construction of siRNA expression plasmids

**Figure 5:**
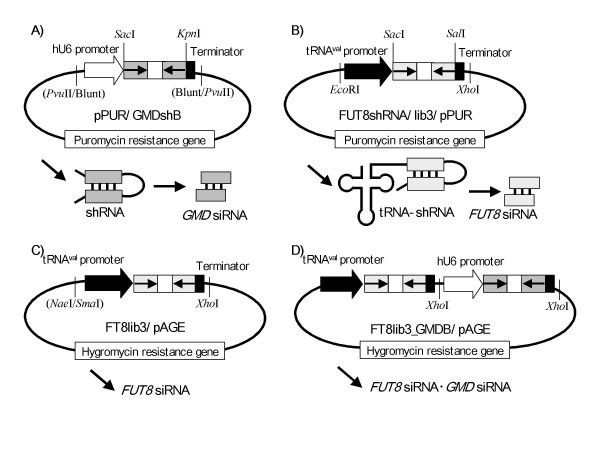
**Structure of siRNA expression plasmids**. The *GMD *siRNA expression plasmid consisted of a puromycin resistance gene and a short hairpin siRNA expression cassette controlled by the human U6 promoter (A). *FUT8 *siRNA expression plasmids consisted of a puromycin or hygromycin resistance gene and a short hairpin siRNA expression cassette controlled by the human tRNA^val ^promoter (B or C). The siRNA tandem expression plasmid consisted of a hygromycin resistance gene and two short hairpin siRNA expression cassettes targeting *FUT8 *and *GMD *(D). The transcribed shRNAs and tRNA-shRNA fusion product were processed into siRNAs by Dicer.

An siRNA expression plasmid for targeting Chinese hamster *GMD *(GenBank: AF525364), pPUR/GMDshB, was generated as described below (Fig. 5A). The plasmid contained a puromycin resistance gene as a selection marker and a *GMD *short hairpin siRNA expression cassette controlled by the human U6 promoter. The fragment of human U6 promoter was prepared by PCR with KOD polymerase (TOYOBO, Tokyo, Japan) from the plasmid U6_FUT8_B_puro [26] using the primers 5'-CCCAAGCTTG ATATCAAGGT CGGGCAGGAA GAGGGCCTAT-3' and 5'-GCTCTAGAGA TATCAAAAAA GGTACCGAGC TCGGTGTTTC GTCCTTTCCA CA-3'. The amplified human U6 promoter was inserted into pPUR (Clontech, Mountain View, CA) at the *Pvu*II site, and the synthetic dsDNA coding short hairpin siRNA against *GMD *(5'-GAGCTCTATA AGAATCCACA GGCTCATATT GAAGGCTTCC TGTCACCTTC AATATGAGCC TGTGGATTCT TATAGGTACC-3') was inserted immediately downstream of the human U6 promoter at the *Sac*I and *Kpn*I sites.

An siRNA expression plasmid for targeting Chinese hamster *FUT8 *[28], FUT8shRNA/lib3/pPUR, contained a puromycin resistance gene as a selection marker and a *FUT8 *short hairpin siRNA expression cassette controlled by the human tRNA^val ^promoter (Fig. 5B). The fragment of human tRNA^val ^promoter was prepared by PCR from the plasmid ptRNA-SS [37] using the primers 5'-TTCCCAGTCA CGACGTT-3' and 5'-CAGGAAACAG CTATGAC-3'. The amplified human tRNA^val ^promoter was inserted into pPUR using the *Pvu*II site, and the synthetic dsDNA coding short hairpin siRNA against *FUT8 *(5'-GAGCTCAAAT CCAAAAGAAT TTCATCTGCA TGTCTTTGGG GATCCCCAAA GACATGCAGA TGAAATTCTT TTGGATTTGT CGAC-3') was inserted immediately downstream of the human tRNA^val ^promoter at the *Sac*I and *Sal*I sites.

Another *FUT8 *siRNA expression plasmid, FT8lib3/pAGE (Fig. 5C), was constructed by insertion of the *FUT8 *short hairpin siRNA expression cassette from FUT8shRNA/lib3/pPUR into pAGE249 [8] as follows. The *FUT8 *short hairpin siRNA expression cassette was excised from FUT8shRNA/lib3/pPUR using the *Eco*RI and *Xho*I sites, and its *Eco*RI terminus was converted to *Sma*I by subcloning into pBlusecriptII KS(+) (Stratagene, La Jolla, CA). The *Sma*I-*Xho*I fragment containing the *FUT8 *short hairpin siRNA expression cassette was inserted into pAGE249 at the *Nae*I and *Xho*I sites to construct FT8lib3/pAGE.

Two siRNA expression plasmids for targeting Chinese hamster GFT (GenBank: AB222037), GFT_3G10/pPUR and GFT_3G10/pAGE, were constructed by replacement of the *FUT8 *dsDNA of FUT8shRNA/lib3/pPUR and FT8lib3/pAGE, respectively, with the dsDNA coding short hairpin siRNA against *GFT *(5'-GAGCTCCCAA AGAGGGTGAG AAGAGTGCTA TTGGGATCCCAATAGCACTC TTCTCACCCT CTTTGGGTCG AC-3').

Finally, an siRNA tandem expression plasmid for the double knockdown of *FUT8 *and *GMD*, FT8lib3_GMDB/pAGE, was constructed by insertion of the *GMD *short hairpin siRNA expression cassette (amplified by PCR from pPUR/GMDshB using the primers 5'-CCGCTCGAGA GCGCCTGATG CGGTATT-3' and 5'-CCGCTCGAGG GACTTTCCAC ACCTGGT-3') into FT8lib3/pAGE at the *Xho*I site (Fig. 5D). In this siRNA tandem expression vector, different PolIII promoters, tRNA^val ^and U6, were designed to prevent unnecessary interference between the two promoter systems.

### Real-Time PCR analyses of *GMD*, *FUT8*, and *GFT*

In order to quantify the amounts of *GMD*, *FUT8*, and *GFT *mRNA in the cells, real-time PCR analysis was carried out using TaKaRa Ex Taq™ R-PCR Version (TaKaRa, Shiga, Japan) and the following four sets of primers: 5'-ATCCTCGTCC TCCTTACTTA CC-3' and 5'-TCCAGCTGAC CAAGAAATAG AG-3' for *FUT8*, 5'-AAGCCCAGGA AGGTGGCGCT CATCAC-3'and 5'-CACTAGTTGA GGCCTGGTAG AACTTCAC-3' for *GMD*, 5'-ATCATCATTG GTGGTTTCTG G-3' and 5'-TCTCTTCATA GTAGAGCACG GC-3' for *GFT*, and 5'-GATATCGCTG CGCTCGTCGT CGAC-3' and 5'-CAGGAAGGAA GGCTGGAAGA GAGC-3' for *β-actin *as an internal standard gene. Total RNA was isolated from 5 × 10^6 ^cells using an RNeasy minikit (Qiagen, Hilden, Germany). Single-strand cDNA was synthesized from 3 μg total RNA using the Superscript™ III first-strand synthesis system for RT-PCR (Invitrogen). A 50-fold diluted reaction mixture was used as a template. PCR was carried out by heating the mixture at 94°C for 5 min followed by 40 cycles of 94°C for 20 s, 65°C for 1 min, and 72°C for 30 s in 20 μL of reaction mixture containing 1 unit of TaKaRa Ex Taq™ R-PCR Version, 1 μL of 2500-fold diluted SYBR Green I (TaKaRa), 5 μL of the diluted single-strand cDNA, and 6 pmol of primers using an ABI PRISM 7700 sequence detection system (Applied Biosystems, Foster City, CA) according to the manufacturer's instructions. After PCR amplification, data acquisition and analyses were performed using the GeneAmp 7700 sequence detection system version 1.7 (Applied Biosystems).

### LCA-staining analysis

Cells (2 × 10^5^) were suspended in phosphate buffered saline (PBS) containing 1% bovine serum albumin (BSA), and either 2 μg/mL fluorescein isothiocyanate (FITC)-labeled LCA (Vector Laboratories, Burlingame, CA) or 2 μg/mL FITC-labeled streptavidin (KPL, Gaithersburg, MD) were added to the suspension. After incubation at 4°C for 30 min, 1 × 10^4 ^stained cells were analyzed by FACScalibur (BD Biosciences, San Jose, CA) to evaluate the reactivity to LCA on the cell surface.

### Analyses of antibody-derived *N*-linked Fc oligosaccharides

Recombinant antibodies were purified from the serum-free culture supernatant by Protein A-affinity chromatography using MabSelect™ (Amersham Biosciences, Piscataway, NJ) and the samples were stored in 10 mM KH_2_PO_4_. The concentration of purified antibodies was measured by absorbance at 280 nm. The monosaccharide composition of each purified IgG1 was characterized by modified high-performance anion exchange chromatography (HPAEC) as previously described [8]. The oligosaccharide profile of each purified antibody was characterized by modified matrix-assisted laser desorption/ionization time-of-flight mass spectrometry (MALDI-TOF MS) in positive-ion mode, as described previously [29].

### Biological activity analysis of antibodies

An ADCC assay for each purified IgG1 was performed by lactate dehydrogenase (LDH) release assay using human peripheral blood mononuclear cells (PBMCs) from healthy donors as effector cells at an E:T ratio of 20:1, as described previously [26]. The antigen-binding activities of the antibodies were measured by antigen-binding ELISA, as previously described [26].

### Isolation of *FUT8*, *GMD*, or *GFT *knockdown clones

An IgG1 antibody-producing clone, CHO/DG44 32-05-12 (1.6 × 10^6 ^cells), was transformed by electroporation with 10 μg of each siRNA expression vector linearized at the *Fsp*I site (pPUR/GMDshB, FUT8shRNA/lib3/pPUR, or GFT_3G10/pPUR). Transfectants were selected in 12 μg/mL puromycin (Sigma-Aldrich) for 7 days, and then the drug-resistant clones were subjected to 7-day selection with 500 μg/mL LCA. The resultant LCA-resistant clones were isolated and expanded in IMDM medium containing 10% (v/v) dFBS, 500 nM MTX, and 12 μg/mL puromycin. Cells were collected for real-time PCR analysis after they had grown to confluence in a tissue-culture flask (Greiner, Frickenhausen, Germany). The serum-free culture supernatants were recovered for antibody analysis after a 7-day culture of the confluent cells in serum-free EX-CELL™ 301 medium (JRH Biosciences, Lenexa, KS).

### Isolation of clones transformed by two siRNA expression vectors

The isolated *GMD *siRNA-introduced clones (1.6 × 10^6 ^cells) were electroporated again with 10 μg of *FUT8 *or *GFT *siRNA expression vector linearized at the *Fsp*I site (FT8lib3/pAGE or GFT_3G10/pAGE). Transfectants were selected in 3 μg/mL puromycin and 400 μg/mL hygromycin (Wako Pure Chemical Industries, Osaka, Japan) for 8 days, and then the drug-resistant clones were subjected to 7-day selection with 500 μg/mL LCA in the presence of 100 μM L-fucose. The resultant LCA-resistant clones were isolated and expanded in IMDM medium containing 10% (v/v) dFBS, 500 nM MTX, 3 μg/mL puromycin, and 400 μg/mL hygromycin for further real-time PCR and antibody analyses, as described above.

### Conversion of antibody-producing cells to cells producing non-fucosylated antibodies

The CHO/DG44 32-05-12 (1.6 × 10^6^) cells were transformed by electroporation with 10 μg of the *FUT8 *and *GMD *siRNA tandem expression vector linearized at the *Fsp*I site (FT8lib3_GMDB/pAGE). Transfectants were selected in 500 μg/mL hygromycin for 8 days, and then the drug-resistant clones were subjected to 7-day selection with 500 μg/mL LCA and 1 mM L-fucose. The resultant LCA-resistant clones were isolated and expanded in IMDM medium containing 10% (v/v) dFBS, 500 nM MTX, and 500 μg/mL hygromycin for further real-time PCR and antibody analyses as described above. The established clones were adapted to serum-free medium EX-CELL™ 302 (JRH Biosciences) supplemented with 6 mM L-glutamine, 500 nM MTX, and 500 μg/mL hygromycin for LCA-reactivity analysis and serum-free fed-batch culture.

### Serum-free fed-batch culture

A 1L-scale spinner bioreactor (ABLE, Tokyo, Japan) was employed to maintain controlled, serum-free, fed-batch culture conditions of 35°C, pH 7.1, 50% DO. Each clone was inoculated at 3.0 × 10^5 ^cells/mL in EX-CELL™302 medium supplemented with 6 mM L-glutamine and 500 nM MTX, and was fed with serum-free IMDM-based feeding medium containing 5.3 μM human recombinant insulin (Invitrogen) at days 3, 5, 7, 9, and 11 post-inoculation to maintain a glucose content of approximately 5.0 g/L. Culture aliquots were drawn on days 3, 6, 9, 12, 14, and 16 prior to addition of the feeding medium. Viable cell density was measured with an automatic cell counter, Vi-CELL™ XR (Beckman Coulter, Fullerton, CA), using trypan blue exclusion. The antibody concentration in the culture supernatant was measured by an enzyme-linked immunosorbent assay (ELISA) specific for human IgG1, as previously described [38].

## Authors' contributions

HI designed the study; participated in the construction of plasmids and the screening of siRNA; carried out cell cultures, transfection, real-time PCR, purification of antibodies; and drafted the manuscript.

KM initiated and worked on the study, and participated in plasmid construction and the screening of siRNA.

MI participated in carrying out the serum-free fed-batch culture.

MW participated in the analyses of antibody-derived *N*-linked Fc oligosaccharides.

SI participated in carrying out the serum-free fed-batch culture, the biological activity analysis, and helped to draft the manuscript.

KS was the head of the laboratories that carried out the present study and also discussed the study from both scientific and industrial points of view.

MS is the head of the laboratories that carried out the present study, initially conceived of the study, and helped to design the study and draft the manuscript.

All authors read and approved the final manuscript.
